# Isolated Gamma-Glutamyl Transferase Elevation in an Asymptomatic Adolescent: An Unusual Presentation of Focal Nodular Hyperplasia

**DOI:** 10.7759/cureus.88050

**Published:** 2025-07-16

**Authors:** Manuel Lima Ferreira, Teresa Cachada Baptista, Diana Alba, Ana Reis

**Affiliations:** 1 Department of Pediatrics and Neonatology, Unidade Local de Saúde Tâmega e Sousa, Penafiel, PRT

**Keywords:** focal nodular hyperplasia, gamma-glutamyl transferase, incidental liver lesion, magnetic resonance imaging, pediatrics

## Abstract

Incidental liver lesions (ILLs) in children are increasingly being identified due to the widespread use of imaging techniques. Focal nodular hyperplasia (FNH) is relatively rare in pediatric age, typically diagnosed based on characteristic imaging findings. We report the case of a 16-year-old asymptomatic female patient with persistent isolated elevation of gamma-glutamyl transferase (GGT), identified during routine evaluation. Further imaging, including abdominal ultrasound and magnetic resonance imaging with hepatocyte-specific contrast agent, revealed an ILL consistent with FNH, despite the absence of a classical central scar. The adolescent remained asymptomatic, and conservative follow-up was decided. This case highlights the diagnostic relevance of isolated GGT elevation as a potential indicator of FNH.

## Introduction

Incidental liver lesions (ILLs) are usually identified in children undergoing abdominal imaging techniques for unrelated reasons. Although most ILLs are benign, their detection may prompt extensive diagnostic workups, potentially leading to invasive procedures and unnecessary anxiety for patients and families [1].

Focal nodular hyperplasia (FNH) is a benign hepatocellular lesion that accounts for approximately 2%-7% of all pediatric liver tumors and represents the second most common benign solid liver lesion in children, following hemangioma [1,2]. It is typically asymptomatic and identified incidentally, with a predominance in female adolescents. The pathogenesis is believed to involve a localized hyperplastic response of hepatocytes to preexisting vascular anomalies, particularly anomalous arterial flow [2,3].

A key challenge in clinical practice is the distinction between FNH and other solitary liver lesions, such as hepatocellular adenoma, hemangioma, regenerative nodules, and malignant tumors such as hepatoblastoma or hepatocellular carcinoma. These entities differ in prognosis and management, and often require different diagnostic and therapeutic approaches. For instance, hepatic adenomas may contain fat or hemorrhagic components and are frequently associated with hormonal exposure, while malignant lesions often present with systemic symptoms and elevation of alpha-fetoprotein [1-3].

In this context, imaging techniques, particularly magnetic resonance imaging (MRI) with hepatocyte-specific contrast agents, play a key role in both diagnosis and follow-up. When characteristic features are present, MRI supports a conservative management strategy, reducing the need for invasive procedures in asymptomatic patients [2,3].

This report describes the case of an asymptomatic adolescent with an incidental diagnosis of FNH following persistent isolated elevation of gamma-glutamyl transferase (GGT), a rare biochemical finding in this context. It highlights the importance of recognizing subtle biochemical abnormalities that may warrant further investigation, particularly in pediatric populations.

## Case presentation

A 16-year-old female patient was referred to the pediatric gastroenterology appointment for persistent and isolated elevation of GGT, documented in two separate blood tests over the course of one year. The initial value was 67 U/L, and a subsequent test one year later showed a further increase to 102 U/L (reference range: 7-32 U/L). The initial laboratory workup was requested by her primary care physician during an unrelated clinical evaluation. No symptoms were reported, including fatigue, abdominal pain, jaundice, pruritus, or weight loss. Her past medical history included asthma, controlled with as-needed bronchodilators. There was no family history of liver or metabolic diseases, and the adolescent was not under regular medication, including hormonal contraception. On physical examination, the adolescent appeared well, with a body mass index of 24 kg/m² (85th percentile for age and sex). Abdominal examination was unremarkable, with no tenderness, hepatosplenomegaly, or palpable masses.

Laboratory workup revealed normal levels of aspartate aminotransferase, alanine aminotransferase, alkaline phosphatase, bilirubin, albumin, total protein, alpha-fetoprotein, and inflammatory markers. Other additional tests, including complete blood count, thyroid function, protein electrophoresis, screening for celiac disease (immunoglobulin A and antitransglutaminase IgA), and serum bile acids, were all within normal limits.

**Figure 1:**
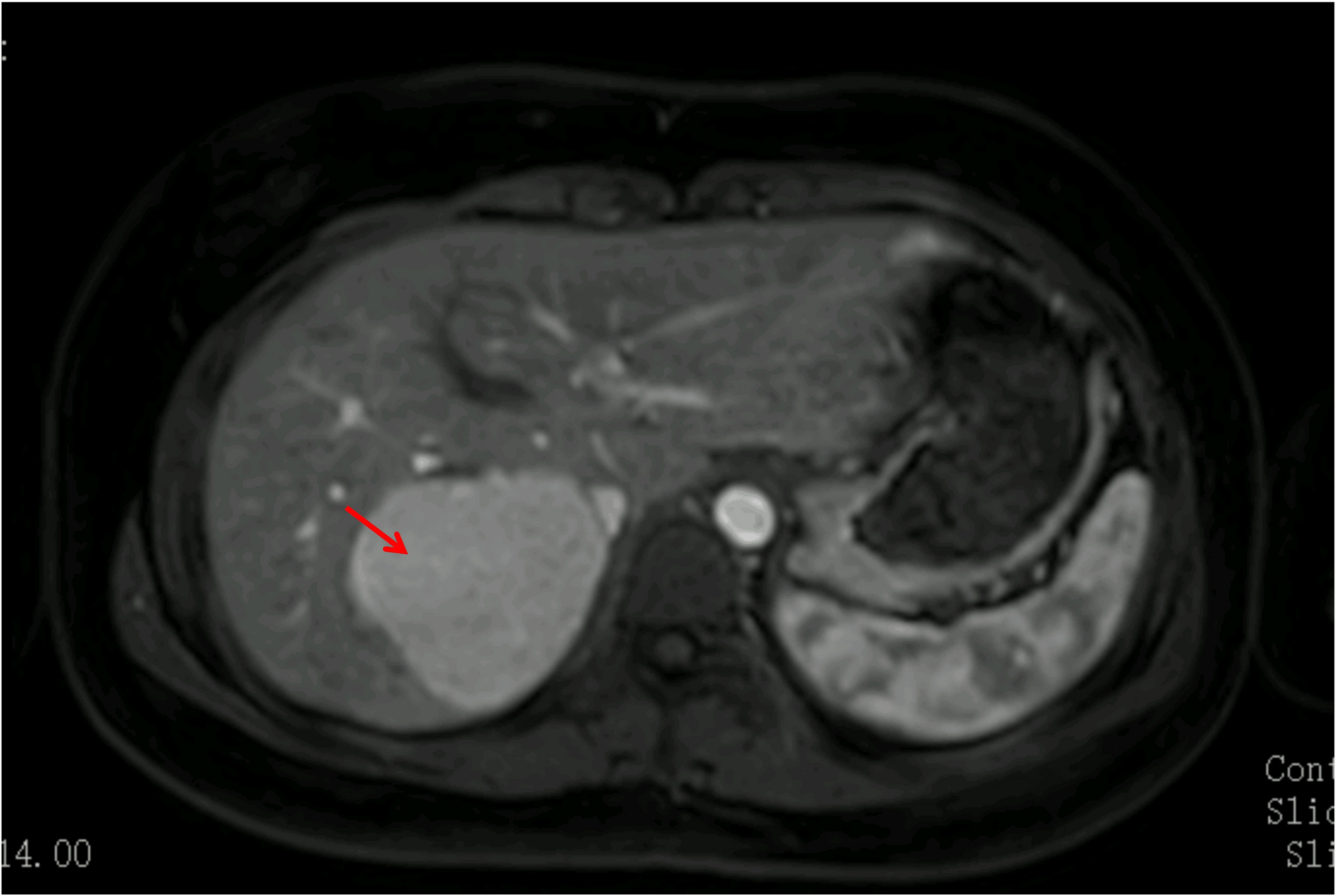
Axial abdominal MRI showing a well-circumscribed lesion in segment VII of the liver (arrow), with homogeneous arterial enhancement MRI: magnetic resonance imaging

**Figure 2:**
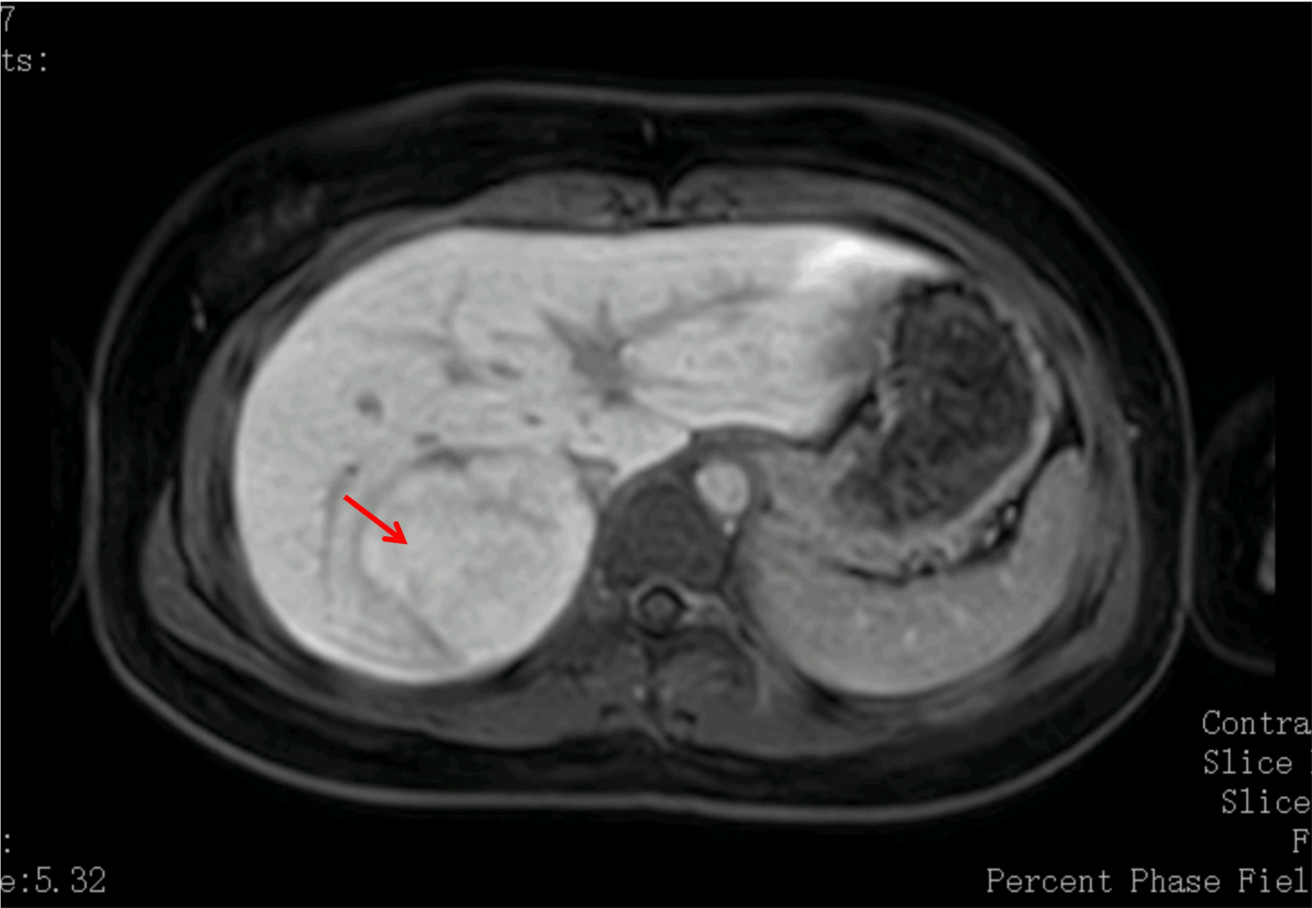
Abdominal MRI with hepatocyte-specific contrast showing a lesion isointense to the surrounding liver parenchyma (arrow), consistent with FNH, with no central scar MRI: magnetic resonance imaging; FNH: focal nodular hyperplasia

Abdominal ultrasound revealed a well-defined, hyperechoic lesion located in segment VII of the liver, associated with mild biliary tract prominence. For further characterization, abdominal MRI with hepatocyte-specific contrast agent was performed, demonstrating a lesion with 6.4 × 5.8 × 6.3 cm, isointense on T1- and T2-weighted sequences, and with intense and homogeneous enhancement during the arterial phase (Figure 1). It was isointense during the venous and hepatobiliary phase, and no central scar was clearly visualized (Figure 2).

The MRI findings were strongly suggestive of FNH. Given the absence of symptoms, typical imaging characteristics, and stable biochemical profile aside from the isolated GGT elevation, a conservative approach was decided, with regular clinical and radiological surveillance. Ten months later, the patient remains asymptomatic and under regular pediatric supervision. No follow-up imaging has been performed to date, but repeat MRI is planned at the one-year mark to reassess lesion stability.

## Discussion

ILLs in children are increasingly being detected due to the wider use of imaging techniques such as ultrasound and MRI. In a recent review, Moreira-Silva et al. highlighted that although each benign lesion is rare, the overall number of incidental findings is growing, and clinicians must balance appropriate investigation with avoidance of overtreatment and unnecessary invasive approach [1]. In this context, FNH represents a diagnostic challenge due to its relative rarity in pediatric age and overlapping features with other liver tumors.

FNH is a benign, nonneoplastic hepatocellular lesion, probably resulting from a localized hyperplastic response to vascular malformations or anomalous arterial flow, without a capsule or risk of malignant transformation [2,3]. In pediatric populations, FNH represents the second most common benign solid liver lesion, typically affecting girls during adolescence [4]. Most patients are asymptomatic, and lesions are discovered incidentally during imaging for unrelated complaints or minor biochemical abnormalities [5].

The differential diagnosis of a solitary liver lesion in a child includes hepatocellular adenoma, regenerative nodules, hemangioma, hepatoblastoma, and hepatocellular carcinoma [6]. Hepatic adenomas are particularly important in adolescents, especially if they are taking hormonal contraception. These lesions often lack hepatobiliary contrast uptake and may contain fat or hemorrhage. Malignant lesions usually present with associated symptoms, elevation of alpha-fetoprotein, or other abnormal laboratory findings [1]. The lack of symptoms, normal laboratory panel (except for GGT elevation), and imaging findings in this case strongly supported a benign diagnosis.

MRI with hepatocyte-specific contrast is the gold standard for noninvasive diagnosis of FNH. Classic features include homogeneous hyperenhancement in the arterial phase, iso- or hypointensity in the portal and delayed phases, and contrast retention in the hepatobiliary phase, due to the presence of functioning hepatocytes [7]. A central stellate scar is classically described, but it may be absent in larger or atypical lesions [5]. This absence should not preclude the diagnosis if the remaining imaging criteria are present. In this case, the lesion was large, well-circumscribed, with the classic contrast enhancement and hepatobiliary uptake, supporting the diagnosis despite the lack of a central scar.

Although most patients with FNH have normal levels of liver enzymes, mild elevations may occasionally be present. GGT elevation has been described in some case series and narrative reviews of FNH, and is thought to reflect subtle biliary compression or altered bile drainage related to lesion size or anatomical location, although this finding remains uncommon and sparsely reported in pediatric populations [8-10]. In this case, mild biliary tract prominence was observed on abdominal ultrasound, suggesting localized disturbance of biliary flow or ductal compression. This radiological finding may help explain the persistent, isolated elevation of GGT over a one-year period, in the absence of other abnormalities or clinical symptoms, further supporting the benign and stable nature of the condition. To our knowledge, no previous case reports have specifically described FNH in pediatric patients presenting solely with isolated GGT elevation.

Due to the absence of malignant potential, FNH does not warrant invasive procedures (such as biopsy or surgical resection) in asymptomatic patients with typical imaging findings. Some authors support clinical and radiological surveillance in pediatric cases, thereby reducing procedural risks and healthcare burden. Follow-up is generally recommended every 6-12 months, particularly during adolescence [1].

This case highlights the diagnostic value of subtle biochemical alterations in prompting further investigation that led to the identification of FNH. It also supports the role of advanced imaging in differentiating benign hepatic lesions and guiding safe, noninterventional management strategies in pediatric patients.

## Conclusions

FNH is a rare, benign, and typically asymptomatic liver lesion in the pediatric population. This case illustrates how persistent and isolated GGT elevation, although often considered a nonspecific finding, may serve as a clinical clue warranting further investigation. When characteristic imaging features are present, particularly on MRI with hepatocyte-specific contrast, a confident diagnosis can be established without the need for invasive procedures. Conservative management with regular clinical monitoring is appropriate and safe in asymptomatic adolescents, minimizing unnecessary interventions and reducing healthcare burden. Further reporting of similar cases may help clarify the significance of isolated GGT elevation in pediatric ILLs.
